# Phylogeny and Classification of *Yersinia pestis* Through the Lens of Strains From the Plague Foci of Commonwealth of Independent States

**DOI:** 10.3389/fmicb.2018.01106

**Published:** 2018-05-25

**Authors:** Vladimir V. Kutyrev, Galina A. Eroshenko, Vladimir L. Motin, Nikita Y. Nosov, Jaroslav M. Krasnov, Lyubov M. Kukleva, Konstantin A. Nikiforov, Zhanna V. Al’khova, Eugene G. Oglodin, Natalia P. Guseva

**Affiliations:** ^1^Russian Research Anti-Plague Institute “Microbe”, Federal Service for Surveillance in the Sphere of Consumers Rights Protection and Human Welfare, Saratov, Russia; ^2^Department of Pathology, University of Texas Medical Branch, Galveston, TX, United States; ^3^Department of Microbiology and Immunology, University of Texas Medical Branch, Galveston, TX, United States

**Keywords:** plague agent, phylogenetic diversity, subspecies classification, PCR and SNP typing, whole genome sequencing

## Abstract

The established phylogeny of the etiological agent of plague, *Yersinia pestis*, is not perfect, as it does not take into account the strains from numerous natural foci of Commonwealth of Independent States (CIS). We have carried out PCR and SNP typing of 359 strains and whole genome sequencing of 51 strains from these plague foci and determined the phylogenetic diversity of the strains circulating here. They belong to 0.ANT3, 0.ANT5, 2.ANT3, 4.ANT branches of antique biovar, 2.MED0, 2.MED1 branches of medieval biovar and to 0.PE2, 0.PE4a. 0.PE4h, 0.PE4t branches. Based on the studies of 178 strains from 23 plague foci of CIS countries, it was determined that the population structure of 2.MED strains is subdivided into Caucasian–Caspian and Central Asian–Chinese branches. In Central-Caucasian high-mountain plague foci in the Russian Federation (RF) the most deeply diverged branch of medieval biovar, 2.MED0, has been found. With the data obtained, the current population structure of *Y. pestis* species has been refined. New subspecies classification is developed, comprising seven subspecies: *pestis, caucasica* (0.PE2), *angolica* (0.PE3), *central asiatica* (0.PE4), *tibetica* (0.PE7), *ulegeica* (0.PE5), and *qinghaica* (0.PE10).

## Significance Statement

The plague pathogen, *Yersinia pestis*, is a striking example of the rapid evolution of highly virulent bacteria that left an indelible mark in the history of mankind. However, the stages of evolution of this bacterium, as well as many aspects of its ecology and pathogenesis remain still unclear. This work contributes to the understanding of the evolution and phylogeography of *Y. pestis* through molecular genetic analysis and whole genome sequencing of strains from plague foci, located in the vast territories of the CIS countries. Genetic diversity of strains, circulating here, is revealed. New populations and previously unidentified phylogenetic branches are found. These data are used to clarify the population structure and improve the intraspecific classification of *Y. pestis*.

## Introduction

Across the territories of the Russian Federation (RF) and other Commonwealth of Independent States (CIS) member-states (former Soviet republics) there are 45 natural plague foci. They are located in a variety of geographical regions – Caspian Sea region, Caucasus, Siberia, and Central Asia, and are situated in high-mountain, mountain, low-mountain, steppe, semi-desert, and desert landscapes. The main carriers of plague are gerbils, ground squirrels, marmots, and small rodents, such as pikas and voles; the arthropod vectors are numerous species of fleas (Onishchenko and Kutyrev, 2004). Diversity of relief patterns, climatic conditions, and natural biocenoses in these plague foci is the cause of diversity of circulating *Yersinia pestis* strains.

According to the accepted classification in RF and other CIS countries, *Y. pestis* strains are divided into five following subspecies (ssp.): main – subspecies (ssp.) *pestis*, and four non-main – ssp. *caucasica*, ssp. *altaica*, ssp. *hissarica*, and ssp. *ulegeica*. The subspecies classification is based on differences in virulence and biochemical activity, as well as in differences of their geographical spread. Strains of the main ssp. are highly virulent. They are widespread in plague foci in various landscape-geographical zones, and cause outbreaks and epidemics of plague. It is these strains that triggered the three devastating plague pandemics in antique and middle ages, as well as in modern times. A distinctive biochemical feature of these strains is the lack of the ability to ferment the disaccharides rhamnose and melibiose. This characteristic is commonly used to differentiate them from strains of non-main ssp. Non-main ssp., in their turn, are primarily virulent for small rodents, but nevertheless some of them are capable of causing plague infection in humans too. All of the non-main ssp. ferment rhamnose and melibiose, but differ in nitrate reduction and arabinose fermentation. These strains are spread throughout a number of plague foci of CIS countries, China and Mongolia.

Strains of the main ssp. were divided earlier into three biovars (bvs.) antique, medieval, and oriental, based on differences in their geographical distribution and in biochemical properties (Devignat, 1951). Strains of medieval biovar (bv.) do not reduce nitrates, strains of oriental bv. do not ferment glycerol, and strains of antique bv. are active against both substrates. Based on the progress in molecular typing technologies and whole-genome sequencing of the strains from different foci around the world, global phylogeny of the plague pathogen has been specified (Achtman et al., 1999; Morelli et al., 2010; Cui et al., 2013). It was established that antique bv. comprises branch 0.ANT, from which other evolutionary branches 1.ANT, 2.ANT, 3.ANT, and 4.ANT of antique bv. originated. Strains of oriental bv. (branch 1.ORI) derived from 1.ANT, and the strains of medieval bv. (branch 2.MED) derived from 2.ANT. Strains of non-main ssp., often called “pestoides,” are the lower branches of the trunk 0, and are designated as 0.PE2–0.PE7.

Achtman et al. (1999) in view of the fact that one of the differential biochemical characteristics – the absence of nitrate reduction – was found in strains of different phylogenetic branches has recommended to group *Y. pestis* strains based on their molecular characteristics rather than biovars. However, it should be emphasized that the CIS countries subspecies classification (in contrast to biovar classification) is based on the analysis of not one but a complex of characteristics and therefore it properly divides the strains in full accordance with their phylogeographical status. Subspecies classification is not contrary to the branch nomenclature system used in scientific publications. In general, biovar classification also does not contradict the branch nomenclature system. It is only important to note that medieval biovar (equivalent to phylogenetic branch 2.MED) comprises only the strains that do not reduce nitrates due to marker mutation in the *napA* gene. An inability to reduce nitrates in some strains of other branches has different genetic grounds and, therefore, they do not belong to medieval biovar. It is also necessary to keep in mind that dividing into biovars (antique, medieval, and oriental) should be applied only to the main ssp., but not to the non-main ssp. Earlier ssp. *caucasic*a was incorrectly placed in antique bv., and ssp. *altaica* was incorrectly placed in medieval biovar. These discrepancies led to the inconsistencies of phenotypic classification of *Y. pestis* strains. For the most part, subspecies classification is more convenient for practical use (e.g., for laboratory diagnostics), than branch nomenclature system. Subspecies classifications are widely used in taxonomy of etiological agents of other dangerous and particularly dangerous infections. At the same time, it should be recognized that subspecies systematics of *Y. pestis* is in need of revision, which should be carried out on the basis of the latest data of the global phylogeny of the plague agent, *Y. pestis.* The adoption of a single subspecies systematics of *Y. pestis* strains is also necessary due to the fact that in recent times different authors use their own systematics, based mainly on the medical significance of subspecies (Li et al., 2009; Platonov et al., 2013; Qi et al., 2016).

Up to this point *Y. pestis* global phylogeny does not include strains from many natural foci of CIS countries. These strains are under-investigated with regard to up-to-date molecular-genetic methods and whole-genome sequencing. We have carried out molecular-genetic analysis of 359 *Y. pestis* strains, applying PCR and SNP typing, as well as whole-genome sequencing of 51 strains, and specified intraspecific and phylogenetic appurtenance of the strains from plague foci of CIS. In this paper we present an expanded global phylogeny and current population structure of *Y. pestis* species through the lens of the strains from the plague foci of CIS countries. New subspecies classification is developed, comprising seven ssp.: *pestis, caucasica* (0.PE2), *angolica* (0.PE3), *central asiatica* (0.PE4), *tibetica* (0.PE7), *ulegeica* (0.PE5), and *qinghaica* (0.PE10). Historical pathways of *Y. pestis* strains dissemination are also discussed.

## Materials and Methods

### *Y. pestis* Strains, Culture Conditions, Biochemical Analysis

This study examined 359 *Y. pestis* strains that were isolated in the plague foci of CIS countries from carriers and vectors of plague, as well as from patients, between 1912 and 2017 (**Table 1** and Supplementary Tables S1, S2). *Y. pestis* strains were received from the State Collection of Pathogenic Bacteria at the premises of the Russian Research Anti-Plague Institute “Microbe” (Saratov, RF). Cultivation of strains and analysis of their biochemical properties was performed in compliance with standard methods of laboratory diagnostics (Onishchenko and Kutyrev, 2013).

**Table 1:** Characterization of *Y. pestis* strains from natural foci of plague of CIS countries.

Countries, foci^∗^, studied strains	Biochemical characteristics^∗∗^	Subspecies, biovar	Phylogenetic branch
Natural plague foci of the Caspian Sea			
Russian Federation, Kazakhstan, Turkmenistan, Uzbekistan, 11 foci (03, 14–20, 23, 26, 43), 89 strains	Rha^-^ Nit^-^Ara^+^ Gly^+^	Main, medieval	2.MED1
Natural plague foci of Caucasus			
Russian Federation, Azerbaidzhan, Armenia, Georgia, 13 foci (01, 02, 04–13, 39), 71 strains	Rha^-^ Nit^-^ Ara^+^ Gly^+^	Main, medieval	2.MED1 2.MED0
	Rha^+^ Nit^+^ Ara^+^ Gly^+^	Ssp. *caucasica*	0.PE2
Natural plague foci of Siberia			
Russian Federation, 3 foci (36–38), 72 strains	Rha^-^ Nit^+^ Ara^+^ Gly^+^	Main, antique	4.ANT 2.ANT3
	Rha^+^ Nit^-^ Ara^-^ Gly^+^	Ssp. *altaica*	0.PE4a
Natural plague foci of Central Asia			
Kazakhstan, Turkmenistan, Uzbekistan, 7 foci (21, 24, 25, 27–30), 39 strains	Rha^-^ Nit^-^Ara^+^ Gly^+^	Main, medieval	2.MED1
Kyrgyzstan, 3 foci (31–33, 35, 40), 77 strains	Rha^-^ Nit^+^ Ara^+^ Gly^+^	Main, antique	0.ANT3 0.ANT5
	Rha^-^ Nit^-^ Ara^+^ Gly^+^	Main, medieval	2.MED1
	Rha^+^ Nit^-^ Ara^-^ Gly^+^	Talas population	0.PE4t
Tadzhikistan, 1 focus (34), 11 strains	Rha^+^ Nit^-^ Ara^-^ Gly^+^	Ssp. *hissarica*	0.PE4h

*^∗^The focus number is given in accordance with the accepted in CIS countries classification of natural plague foci (Onishchenko and Kutyrev, 2004).*

*^∗∗^Rha – rhamnose fermentation, Nit – nitrate reduction, Ara – arabinose fermentation, Gly – glycerol fermentation.*

### PCR- and SNP- Typing

To identify intraspecific and phylogenetic appurtenance of *Y. pestis* strains we used a system of molecular typing methods based on PCR and SNP assays. PCR assay was conducted employing indel mutations that are the markers for different *Y. pestis* ssp., bvs, phylogenetic branches (Supplementary Table S3A). Two target sequences MED24 and glpD, were applied to differentiate between the bvs. of the main ssp. (Motin et al., 2002; Eroshenko and Kutrev, 2012). To distinguish the strains of ssp. *pestis* (main ssp.), mutations in *gptB-yoaE* and *ilvB-ilvN* loci were identified, where strains of the main ssp. show 89 and 45 bp deletions, respectively. To differentiate between the non-main ssp., the marker mutations were searched as follows: for ssp. *caucasica* – 91 bp deletion in YPO0445 gene, ssp. *ulegeica* – 88 bp deletion in the intergenic region between YPO1452 and YPO1453, ssp. *hissarica* – 205 bp deletion in YPO2267, and for Talas strains – 72 bp deletion in YPO2412, microtus strains – 112 bp deletion in *araC* gene (designation of genes are given as in the genome of reference strain CO92, Accession No. NC_003143.1 in NCBI GenBank). To separate *Y. pestis* strains of two phylogenetic branches 1 and 2 from each other, the DNA targets designated as 1.ANT/1.ORI and 2.ANT/2.MED were detected (Nikiforov et al., 2015). In strains of branch 1, 1.ANT/1.ORI contains a part of the cusφ phage sequence, which is absent in other *Y. pestis* strains. The latter one, 2.ANT/2.MED, in strains of branch 2 contains a marker deletion that is the size of 70 bp, and 4.ANT strains were detected using the region of pTP33 plasmid, found in all the strains of this branch.

The target DNAs were amplified in 25 μl reaction mixture containing 1x PCR buffer (Invitrogen), 1.5 mM MgCl_2_, 0,2 mM dNTPs (Invitrogen), 10 pM primer (Syntol, Russia) and 1 U Taq DNA polymerase (Invitrogen). Amplification of target DNAs required an initial 5 min denaturation at 94°C and 35 cycles of 45 s denaturation at 94°C, 30 s annealing at 55–58°C, and a 45 s extension at 72°C followed by a final 3 min extension at 72°C.

Intraspecific differentiation was carried out based on the size of amplifiable loci or presence/absence of these loci (Supplementary Table S3A). *Y. pestis* strains typical to different intraspecific populations were used as a positive control when conducting PCR to avoid false negative results.

For identification of the appurtenance of *Y. pestis* strains to certain phylogenetic branches, marker SNPs were found (Supplementary Table S3B). The search of the SNPs was conducted with Wombac v2.1 software^1^ by comparing the whole-genome sequences of *Y. pestis* strains of different phylogenetic branches. For SNP typing, SNPs were amplified in PCR (95°C, 5 min; 35 cycles: 95°C – 45 s, 54°C – 40 s, 72°C – 45 s; 72°C – 3 min), then sequenced and aligned against the genome of the reference strain *Y. pestis* CO92 to identify single nucleotide substitutions. Sequencing of PCR fragments was performed in Genetic Analyzer ABI PRISM 3500XL (Applied Biosystems). For each phylogenetic branch, one or two marker SNP’s were used.

### Whole-Genome Sequencing, SNPs’ Identification, Dendrogram Graphing

Whole-genome sequencing of *Y. pestis* strains was carried out in Ion PGM system (Life Technologies). The data processing and raw short-read sequences assembling *de novo* were accomplished using Ion Torrent Suit software package 4.4.2 and Newbler gsAssembler 2.6. The sequence reads were assembled into genomes, resulting in average coverage per genome of 98, 96% (52, 85-fold depth) and an average genome assembly size of 4,59 Mb.

Core SNPs were identified by aligning contigs of *Y. pestis* strains to CO92 genome through Wombac 2.0 software program, then, 28 homoplastic SNPs were excluded (Cui et al., 2013) (Supplementary Tables S4, S5).

The Maximum likelihood tree was constructed using software PHYML, HKY85 model and 500 bootstrap replications. The Maximum parsimony tree was constructed in Bionumerics 7.6.

## Results

**FIGURE 1:**
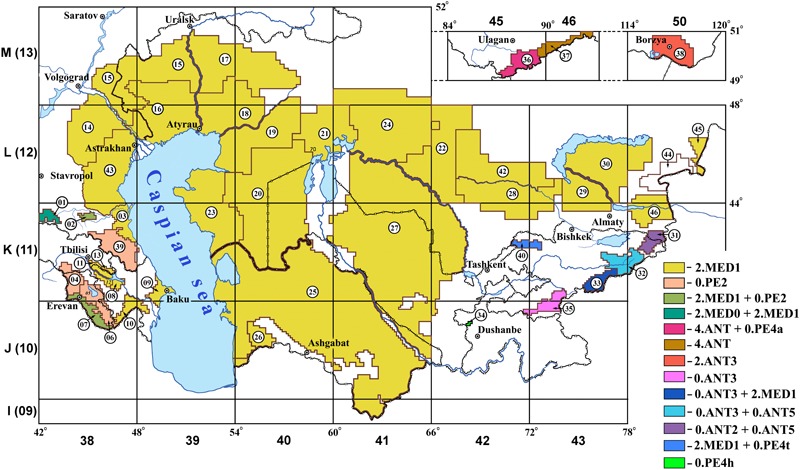
Phylogenetic appurtenance of *Yersinia pestis* strains from CIS plague foci. The index number corresponds to the classification of foci, applied in CIS countries.

**FIGURE 2:**
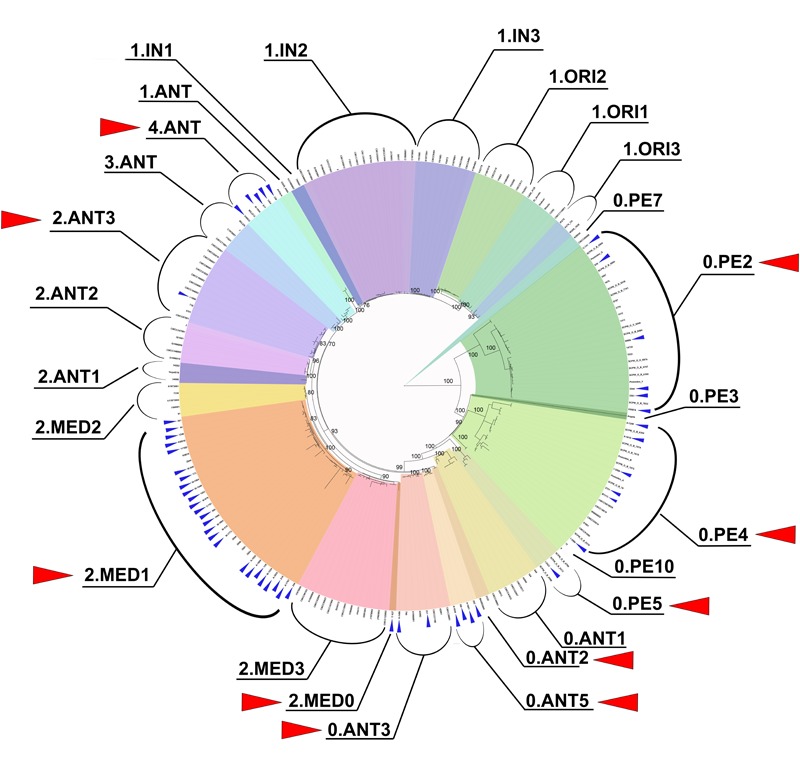
Phylogenetic diversity of *Y. pestis* strains from CIS plague foci, revealed by core genome SNP analysis. Red triangles mark phylogenetic branches, the strains of which circulate in the plague foci of the CIS countries. Small blue triangles mark strains, sequenced by us. Maximum likelihood tree of 207 *Y. pestis* genomes, based on 1708 SNPs, is constructed using PHYML 3.1 with the HKY85 model and 500 bootstrap replications.

We have carried out complex analysis of molecular-genetic and phenotypic properties of 359 *Y. pestis* strains from CIS plague foci (**Table 1**) and sequenced 51 genomes out of these strains (Supplementary Tables S1, S2). Global phylogenetic analysis covered whole-genome sequences of these 51 strains and of 156 *Y. pestis* genomes, obtained from NCBI GenBank (Supplementary Table S4), including genomes from CIS foci sequenced by other researchers (Kislichkina et al., 2015; Zhgenti et al., 2015). As a result, we specified the position of strains from CIS plague foci in global phylogeny of *Y. pestis* (**Figures 1, 2** and **Table 1**).

To determine intraspecific and phylogenetic relations of the studied *Y. pestis* strains, we have used a system of molecular typing based on PCR and SNP assays, employing indel and SNP mutations specific to different *Y. pestis* ssp., bvs., and phylogenetic branches (Supplementary Tables S3A,B). In our experiments we have observed full agreement between the phylogenetic assignments of *Y. pestis* strains of all studied lineages, based on PCR, and SNP typing and whole genome sequencing.

### *Y. pestis* Strains From the Natural Plague Foci of the Caspian Sea Region – RF, Kazakhstan, Turkmenistan, Uzbekistan

**FIGURE 3:**
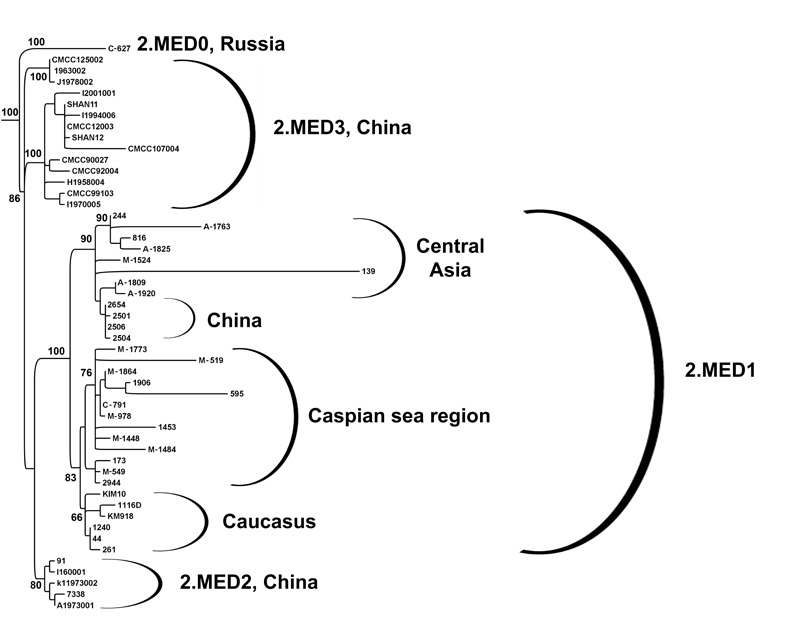
Phylogenetic analysis of *Y. pestis* strains of 2.MED branch of medieval bv. from plague foci of CIS and neighboring countries (fragment of the global phylogeny dendrogram of *Y. pestis*, shown in **Figure 2**). Maximum likelihood tree, PHYML 3.1 software program, HKY85 model and 500 bootstrap replications. Strains M-1864 (43), M-978 (14), M-1773 (16), M-1484 (15), M-1906 (43), M-595 (43), C-791 (03), M-1448 (17), M-1453 (18), 173 (23), M-549 (20), and M-519 (26) are from plague foci of the Caspian Sea region of RF, Kazakhstan, Uzbekistan, and Turkmenistan; strains KM918 (01), KM919 (01), 1116D (02) 44 (08), 1240 (10), 261 (09) – from foci of Caucasus of RF, Azerbaidzhan; strains 244 (21), KM816 (25), A-1763 (24), 139 (29), A-1825 (27), M-1524 (23), A-1809 (40), A-1920 (30) – from foci of Central-Asian region (Kazakhstan, Uzbekistan, and Turkmenistan); other strains (NCBI GenBank) are from plague foci of China and Iran (strain KIM10). Each designation consists of the strain name followed by the number of plague foci in which they were isolated in parenthesis.

Eleven natural plague foci are spread across the territory of the Caspian Sea region (**Figure 1** and **Table 1**). They are situated in RF, Kazakhstan, Turkmenistan, and Uzbekistan. We have investigated 89 *Y. pestis* strains that were isolated in the plague foci of Caspian Sea region from carriers and vectors of plague, as well as from patients, between 1912 and 2014. PCR and SNP assays of these strains has established that they fall into medieval bv., branch 2.MED1. Their genome contains the marker for the medieval bv. 24 bp deletion, as well as the marker for phylogenetic branch 2.MED1 SNP in YPO2744 locus (Supplementary Tables S3A,B). Whole-genome sequencing of 12 *Y. pestis* strains M-1864, M-978, M-1773, M-1484, 1906, M-595, C-791, M-1448, M-1453, 173, M-549, and M-519 (Supplementary Table S1), has confirmed that they belong to the 2.MED1 branch. Phylogenetic analysis of these strains showed that they exist in a single sub-cluster, which forms a common cluster with another sub-cluster of 2.MED1 strains, isolated in plague foci of the Caucasus (**Figure 3**). We have designated this cluster as the Caucasian–Caspian branch of 2.MED1. Strains of other phylogenetic branches of *Y. pestis* were not found in the Caspian Sea region.

### Natural Plague Foci of Central Asia – Kazakhstan, Uzbekistan, and Turkmenistan

We have studied 39 strains from 7 plague foci of Central Asian region: Kazakhstan, Uzbekistan, and Turkmenistan. We completed whole-genome sequencing of strains *Y. pestis* 244, KM816, A-1763, 139, A-1825, M-1524, and A-1920 from these plague foci. By reference to the set of evaluated characteristics, all the strains are similar to each other and fall into phylogenetic branch 2.MED1. On the dendrogram these strains come in a single sub-cluster, which forms common cluster with another sub-cluster of 2.MED1 strains, isolated in China (**Figure 3**). We have designated this cluster as the Central Asian – Chinese branch of 2.MED1 strains.

### Natural Plague Foci of Caucasus – RF, Azerbaidzhan, Armenia, Georgia

Thirteen plague foci are spread across the Caucasus. They are situated in RF, Azerbaidzhan, Armenia, and Georgia (**Figure 1** and **Table 1**). We have studied 71 strains from these foci, which were isolated from carriers and vectors between 1970 and 2003. Assessment of their properties, including PCR and SNP typing, showed that in nine plague foci circulating *Y. pestis* strains belong to 2.MED1 branch of medieval bv. Whole-genome sequencing of *Y. pestis* strains KM918 and 1116D (from plague foci of the Caucasus in RF) and strains 1240, 261, and 44 (from plague foci of Azerbaidzhan) testified to the fact that they fall within the 2.MED1 branch. These strains come in a single sub-cluster and together with sub-cluster of strains from the Caspian Sea foci form common cluster, which we have designated above as a Caucasian–Caspian branch of 2.MED1 (**Figure 3**).

At the same time, a number of strains from Central-Caucasian high-mountain focus (01) that related to 2.MED branch of medieval bv. did not belong to the 2.MED1 branch or the other two known 2.MED2 and 2.MED3 branches of medieval bv., as these strains did not contain marker for nucleotide substitutions in YPO2744, YPO1299, and YPO0652 (Supplementary Table S3B). They were isolated in the Western and Central parts of Central-Caucasian high-mountain focus. Whole-genome sequencing of one of the strains, *Y. pestis* KM919, has revealed its appurtenance to a different population of the 2.MED branch, earlier not exemplified in the global phylogeny of *Y. pestis*. These strains belong to the most deeply diverged branch of medieval bv., which occupy an intermediate position between 2.ANT and 2.MED strains. We have designated that newly found branch as 2.MED0 (**Figures 2, 3**).

In 6 of the 13 foci in the Caucasus, *Y. pestis* strains of 0.PE2 branch (ssp. *caucasica*) circulate. This branch is one of the deepest branches of *Y. pestis*. Whole-genome sequencing of the strain C-741 from RF and strains 835, 3551, 3544, KM874, and M-986 from other Caucasian foci has shown that *Y. pestis* strains from East-Caucasian high-mountain focus in RF (strain C-741) belong to the deepest line of 0.PE2 strains (**Figure 2**).

### Natural Foci of Siberia, RF

There are three natural plague foci in Siberia: Gorno-Altay high-mountain (36), Tuva mountain (37), and Trans-Baikal steppe (38) foci (**Figure 1**). We have investigated 72 *Y. pestis* strains from the foci of Siberia, which were isolated between 1923 and 2017. All the strains from Trans-Baikal steppe, Tuva mountain foci, and part of the strains from Gorno-Altay high-mountain focus fall into antique bv. The genome of the strains from Trans-Baikal steppe focus contained a marker for phylogenetic 2ANT/2MED branch 70 bp deletion and a marker for phylogenetic branch 2.ANT3 nucleotide substitution in the YPO3506 locus (Supplementary Tables S3A,B). Whole-genome sequencing of *Y. pestis* KM682 has confirmed that the strains from this focus belong to 2.ANT3 branch. Similar strains circulate in the bordering Khenteisky aimak in Mongolia, as well as in border-line territories of China (Kukleva et al., 2015).

All strains from the Tuva mountain focus and part of the strains from the Gorno-Altay high-mountain focus belong to the 4.ANT branch, as they contain the marker SNP in the YPO1418 locus, as well as the pTP33 plasmid sequence, found in strains of the 4.ANT branch (Afanas’ev et al., 2016). Whole-genome sequencing of the strains KM932 (I-3223), M-1944, 1454, 517, and 338 from these two foci confirmed their appurtenance to the 4.ANT branch.

**FIGURE 4:**
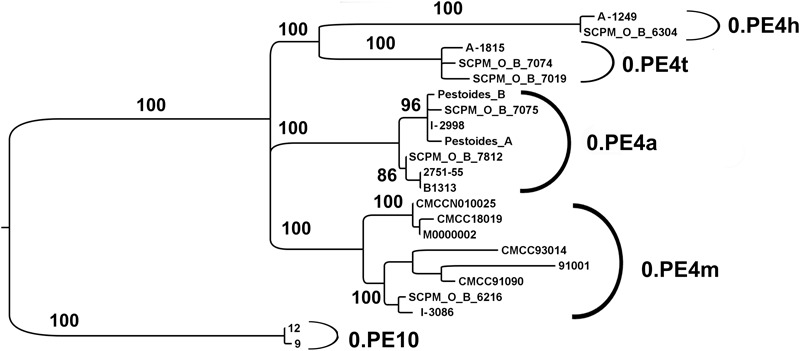
Phylogenetic analysis of *Y. pestis* strains of 0.PE4 branch from natural plague foci of RF, Tadzhikistan, Kyrgyzstan, Mongolia, and China countries (fragment of the global phylogeny dendrogram of *Y. pestis*, shown in **Figure 2**). Maximum likelihood tree, PHYML 3.1 software program, HKY85 model and 500 bootstrap replications. Strains I-2998, 2751, and B1313 are from the Gorno-Altay high-mountain focus (36); A-1249 from Hissar high-mountain focus (34); A-1815 from Talas high-mountain focus (40); I-3086 from Mongolia; other strains are from NCBI GenBank: SCPM_O_B_7075, SCPM_O_B_7812 from Gorno-Altay high-mountain focus (36); SCPM_O_B_6304 from Hissar high-mountain focus (34); SCPM_O_B_7074, SCPM_O_B_7019 from Talas high-mountain focus (40); Pestoides A and Pestoides B – from CIS countries, other strains are from China.

The strains of the second *Y. pestis* population from the Gorno-Altay high-mountain focus pertain to the 0.PE4 cluster. They contain a 90 bp deletion in YPO1226, which is characteristic to ssp. *altaica.* Based on the results of whole-genome sequencing, strains I-2998, I-2751, and B1313 have been assigned to the 0.PE4 cluster. These strains form a separate group in the dendrogram together with other altai strains, the genomes of which are deposited in NCBI GenBank (**Figure 4**). We have designated this branch as 0. PE4a (altai strains).

### Natural Plague Foci of Tien Shan and Pamir-Alay – Kyrgyzstan, Tadzhikistan

**FIGURE 5:**
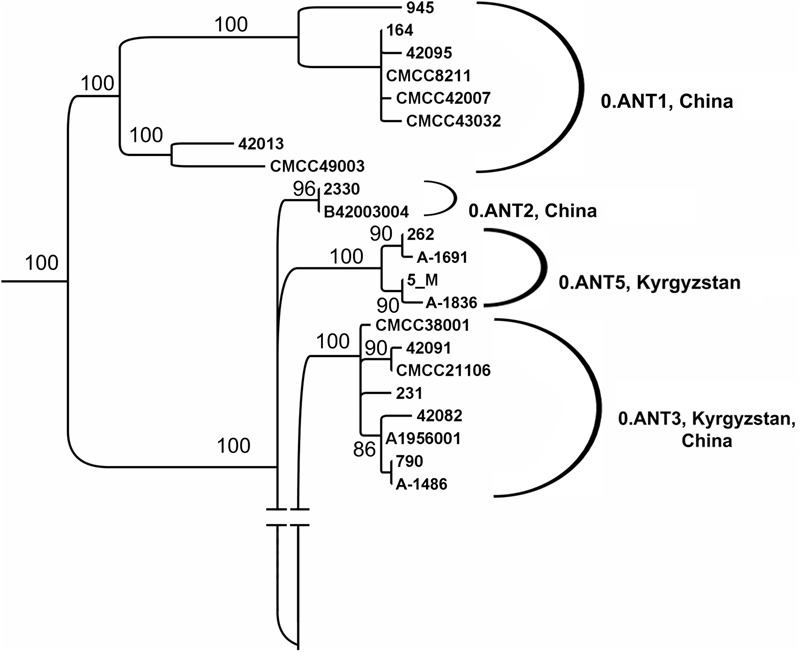
Phylogenetic analysis of *Y. pestis* strains of antique bv., branch 0.ANT from plague foci of Kyrgyzstan (fragment of the global phylogeny dendrogram of *Y. pestis*, shown in **Figure 2**). Maximum likelihood tree, PHYML 3.1 software program, HKY85 model and 500 bootstrap replications. Strains: A-1836, A-1691, 231, A-1486, 5M, and 262 are from Tien-Shan and Alay high-mountain foci; other strains (NCBI GenBank) are from China, strain 790 is from Kyrgyzstan.

Three plague foci are situated in Kyrgyzstan: Tien-Shan (31–33), Alay (35), and Talas (40) high-mountain foci. In total, 77 *Y. pestis* strains isolated between 1928 and 1983 were analyzed. Sixty six strains were isolated in the Tien-Shan and Alay high-mountain foci. They belong to the main ssp. Three strains are incapable of nitrification and denitrification and contain marker 24 bp deletion and marker SNP in the YPO2744 locus, therefore, belong to phylogenetic branch 2.MED1. Other strains have been classed as ancient branch 0.ANT of antique bv. using PCR and SNP typing. We have detected 0.ANT3 strains, containing SNP in YPO1758 locus (Supplementary Tables S3A,B). However, we were unable to assign a large number of strains using the SNPs, marker for 0.ANT1, 0.ANT2, and 0.ANT3 branches. Whole-genome sequencing of four of these strains, A-1691, A-1836, 5M, and 262 has been performed, which made it clear that these strains form a separate branch of 0.ANT strains not exemplified earlier in the global phylogeny of *Y. pestis*. We have designated it as 0.ANT5 (**Figure 5**). The other non-identified 0.ANT strains from the Tien-Shan high-mountain focus also belong to the new 0.ANT5 branch. They carry two marker SNPs in YPO114 and YPO01105 loci, which we have found to be specific to the 0.ANT5 branch.

According to PCR and SNP typing, out of the 11 *Y. pestis* strains from another focus of Kyrgyzstan (Talas high-mountain focus) one strain belongs to the 2.MED1 branch of medieval bv. The other 10 strains are related to the 0.PE4 cluster. Whole-genome sequencing of one of the Talas *Y. pestis* strains, A-1815, showed that this strain together with two other Talas strains from NCBI GenBank form a separate branch within the 0.PE4 cluster. Therefore, we have named the Talas strains as 0.PE4t (Talas strains). As a result, the diversity of *Y. pestis* strains circulating in the plague foci in Kyrgyzstan, falling under 0.ANT3, 0.ANT5, 2.MED1, and 0.PE4t branches has now been determined.

Hissar high-mountain focus (34) is situated in Tadzhikistan on the border with Kyrgyzstan. Eleven *Y. pestis* strains isolated in 1970–1972 were investigated. All the strains demonstrated similarity in characteristic properties and carried the specific Hissar ssp. 205 bp deletion in the YPO2267 gene. Based on the results of whole-genome sequencing, *Y. pestis* strain 1249 has entered the 0.PE4 cluster. We have designated Hissar strains as 0.PE4h (**Figure 4**).

## Discussion

*Yersinia pestis* caused three plague pandemics over the course of history: during the antique period (Plague of Justinian, 541–543), the medieval period (Black Death, 1347–1353), and in modern times (began in China in 1855). Initially, it was considered that the first plague pandemic took origin in North Africa, while the second pandemic was imported into Europe from the Caspian Sea coast. Later on, in view of the discovered diversity of *Y. pestis* strains in the foci of China, it was posited that the plague pathogen had originated in China and that all the three historical plague pandemics emerged due to importation of antique, medieval and oriental bv. strains from China foci to other continents during the military campaigns of Chinese military commanders, as well as via the Silk Route in different historical epochs (Morelli et al., 2010; Cui et al., 2013). This hypothesis became widespread mainly due to the lack of data on *Y. pestis* strains disseminated in other numerous plague foci of Eurasia and especially of CIS countries in the modern period, as well as in the past historical era. Impressive advances in studies of archeogenomes from burial sites dated back the times of the first and the second pandemics of plague, as well as our research data on the phylogenetic diversity of strains from CIS plague foci allowed us to view the current population structure of *Y. pestis* species and historical pathways of its dissemination in a new light. Rasmussen et al. (2015) have demonstrated that the most ancient *Y. pestis* strains existed in the territory of Altay as far back as the Bronze Age, and those strains are the predecessors of all present-day plague agent strains. Valtueña et al. (2017) have shown that the most ancient known *Y. pestis* genomes are from the Caucasus (Rasshevatskiy, Russia) and South Siberia. These data call into question the hypothesis on *Y. pestis* origination in China. Furthermore, reconstruction of ancient *Y. pestis* genomes from the burial sites of the first plague pandemic established that these strains relate to the 0.ANT phylogenetic branch, and are positioned between modern 0.ANT1 and 0.ANT3 populations. They are designated as 0.ANT4 (Harbeck et al., 2013; Wagner et al., 2014). We have obtained data testifying to the fact that 0.ANT strains (0.ANT3 and newly found 0.ANT5) are widely spread in the high-mountain foci of Tien Shan and of North-Eastern part of Pamir-Alay in Kyrgyzstan (**Figure 5**). It suggests that antique 0.ANT strains are indigenous to high-mountain foci of Tien Shan and Pamir-Alay and that the first plague pandemic could have originated in these foci, where 0.ANT strains are widely spread.

Reconstruction of ancient genomes from the burial sites of the second plague pandemic positioned them at the base of 1.ANT branch. This was substantiated by studies of more recent burials of the period of the second pandemic, in the territory of Tatarstan, RF. These studies demonstrated that the natural plague focus persisted in the territory of medieval Europe over a period of several centuries provided for the recurrent manifestation of the disease within that period (Bos et al., 2016; Seifert et al., 2016; Spyrou et al., 2016). It was proposed that strains of the 1.ANT branch, which triggered the second plague pandemic, reached the territory of China. This gave rise to 1.ORI strains that ignited the third pandemic of plague.

Strains of the medieval bv. became widespread until the late middle ages. Publications on *Y. pestis* strains of medieval bv. are few in numbers. Our study somewhat closes this gap by investigating 178 strains of medieval biovar from 23 plague foci of CIS countries and sequencing of 26 of these strains. We found that strains of branch 2.MED1 are widely disseminated in the plague foci of the Caspian Sea region, Caucasus and Central Asia. The 2.MED1 strains are clearly subdivided into two branches, Caucasian–Caspian and Central Asian–Chinese, in accordance with the geographical regions of their distribution (**Figure 3**). Yet, in the Central-Caucasian high-mountain focus in RF, medieval strains of the most deeply diverged 2.MED0 branch have survived. They possess a number of unique characteristics, including presence of an additional plasmid pCKF that codes proteins of transport and of the type 4 secretion system (Oglodin et al., 2015). The persistence of the deepest branch of medieval bv. in this region suggests that the hypothesis that medieval strains could have originated near the Caspian Sea may prove to be correct.

This paper also expands the diversity of 0.PE4 branch strains. Using the results of whole- genome sequencing of the strains from the CIS plague foci, the current population structure of the 0.PE4 branch is defined (**Figure 4**). On the dendrogram, the 0.PE4 strains form a separate cluster of *Y. pestis*, which clearly splits into four sub-clusters: 0.PE4a (ssp. *altaica*, RF, Mongolia), 0.PE4m (microtus strains from two foci in China), 0.PE4h (ssp. *hissarica*, Tadzhikistan), and 0.PE4t (Talas strains, Kyrgyzstan). The 0.PE4 cluster also includes a separate sub-cluster of strains from the Qinghai province in China. It is important to note that 0.PE4a, 0.PE4h, 0.PE4t, and 0.PE4m strains of the 0.PE4 branch share similar characteristics. For example, they ferment rhamnose and glycerol, do not reduce nitrates, do not ferment arabinose and unlike other strains of *Y. pestis* do not cause plague in humans (Onishchenko and Kutyrev, 2004; Cui et al., 2013). We suggest consolidating the strains of the 0.PE4 branch into one novel ssp., naming it ssp. *central asiatica*, taking into account their similar characteristics, as well as their spread in the Central Asian zone of natural plague focality. Strains of the deeply diverged and deviant Qinghai sub-cluster, in contrast, have differing biochemical characteristics and can cause plague in humans. These strains have only a few SNPs common to other strains of the 0.PE4 branch and a lot of their own unique SNPs, therefore, at large they do not belong to the 0.PE4 branch. Instead, they should be related to the individual phylogenetic branch (designated as 0.PE10) and to a separate subspecies – *ssp. qinghaica*.

### Current Population Structure of *Y. pestis* Species and Enhancement of Its Intraspecific Classification

**FIGURE 6:**
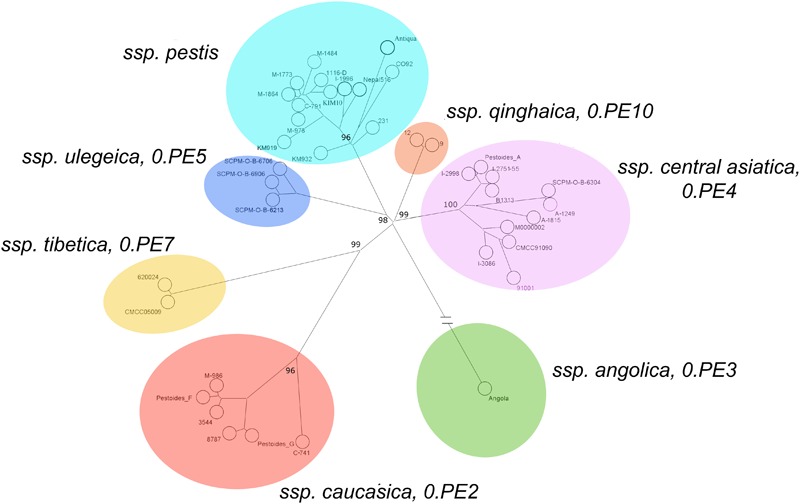
Current population structure of *Y. pestis* revealed by core genome SNP analysis (based on 2413 core SNPs of 39 genomes of strains, belonging to different phylogenetic branches and populations), and enhancement of subspecies classification of *Y. pestis*. Maximum parsimony tree, Bionumerics 7.6 software program.

The data obtained in this study on *Y. pestis* strains from CIS plague foci expand the idea of global phylogenetic diversity of this bacterial species (**Figure 2**). **Figure 6** shows the current population structure of *Y. pestis*, built for clarity upon whole-genome SNPs assay of 39 strains belonging to different phylogenetic branches and populations (Supplementary Table S5).

All *Y. pestis* strains in the dendrogram split into seven separate clusters. The largest cluster comprises strains of the main ssp. of antique, medieval and oriental bvs. Strains of ssp. *caucasica* (0.PE2) form another distinct cluster, as well as two strains from Chinese Tibet (0.PE7). A distant cluster is constituted by the Angola strain from Africa (0.PE3), and another distinct cluster is composed of strains of the most recent non-main ssp., ssp. *ulegeica* from Mongolia (0.PE5). At the same time, all the strains of the altai ssp. and the hissar ssp., along with the Talas strains from Kyrgyzstan and microtus strains from China, merge into one common cluster. This proves their appurtenance to a common branch of evolution detached from other branches of *Y. pestis*. All of the strains of the branch 0.PE4 split into four clusters: 0.PE4a, 0.PE4m, 0.PE4h, and 0.PE4t. Two strains from the Qinghai province of China form a separate cluster, designated as (0.PE10), that is distinct from other 0.PE4 strains.

On the basis of the identified population structure of *Y. pestis* strains, we suggest the following intraspecific taxonomy of the present-day plague agent strains, which includes seven ssp.: *pestis* (bvs: antique, medieval, oriental, and possibly, intermedium too), *ssp. caucasica –* 0.PE2, *ssp. angolica* – 0.PE3, ssp. *central asiatica* (bvs: altai, microtus, hissar, talas) – 0.PE4, ssp. *tibetica* – 0.PE7, ssp. *ulegeica* – 0.PE5, and ssp. *qinghaica* – 0.PE10. From the dendrogram (**Figure 6**) it is clear that microtus strains merge with the microtus sub-cluster of *ssp. central asiatica* and cannot be designated as an individual ssp. The given population structure of *Y. pestis* species clearly demonstrates that it is also incorrect to place all non-main ssp. into microtus ssp., since the non-main ssp. pertain to different phylogenetic branches. This incorporation is of artificial nature (Platonov et al., 2013; Qi et al., 2016).

Overall, on the basis of complex analysis of 359 *Y. pestis* strains and whole-genome sequencing of 51 strains from the natural plague foci of CIS countries, we have identified their intraspecific and phylogenetic appurtenance. Strains of the main ssp. of antique bv. (phylogenetic branches 0.ANT3, 0.ANT5, 2.ANT3, 4.ANT), medieval bv. (2.MED0, 2.MED1 branches), and of non-main ssp. (branches 0.PE2, 0.PE4a. 0.PE4h, 0.PE4t) have been identified. This work also developed a new subspecies classification of *Y. pestis*, reflecting its current present-day population structure.

## Author Contributions

VK, GE, and VM designed research, analyzed the data, and wrote the paper. GE, NN, JK, LK, KN, ZA, EO, and NG performed the research. The authors contributed equally to this work.

## Conflict of Interest Statement

The authors declare that the research was conducted in the absence of any commercial or financial relationships that could be construed as a potential conflict of interest. The reviewer AA declared a past co-authorship with one of the authors VM to the handling Editor.

## Footnotes

1 https://github.com/tseemann/wombac
