# Supplementary material for: Phylogeny and Classification of Yersinia pestis Through the Lens of Strains From the Plague Foci of Commonwealth of Independent States
Source: Front Microbiol. 2018 May 25;9:1106. doi: 10.3389/fmicb.2018.01106 (PMC5980970; doi:10.3389/fmicb.2018.01106)
Supplement: Supplementary file 3 [file Table_3.docx]

**Table S3*A*. DNA targets and primers used for intraspecific differentiation of *Y. pestis* strains by PCR**

| DNA target | Marker characteristic | Primer sequences  5’→3’ | Localization within the genome *Y. pestis*С092* | Size of amplifiable locus (bp) or  presence + (bp)/ absence –  of amplifiable focus | References |
| --- | --- | --- | --- | --- | --- |
| 1 | 2 | 3 | 4 | 5 | 6 |
| MED24 | 24 bp deletion in the strains of the main subspecies, medieval biovar | S – GTATTTTGTGTCACCCC  As – AATGAGACACCGCCAGT | 996412-996633 | Strains of the medieval biovar, 198  Other strains, 222 | Eroshenko and Kutyrev, 2012 |
| glpD | 93 bp deletion in the strains of the main subspecies, oriental biovar | S – CAGAGGAAGGTAACATGGA  As – CAAGGCTTCACTTACCAAG | 4421252-4421370 | Strains of the oriental biovar, 415  Other strains 508 | –“– |
| gptB-yoaE | 89 bp deletion in the strains of the main subspecies | S – GCTGCGTATCATTTCACC  As – AATCAAATCTCGCCCAGC | 2004340-2004639 | Strains of the main subspecies, 300  Other strains, 389 | –“– |
| ilvB-ilvN | 45 bp deletion in the strains of the main subspecies | S – AGTGGTCTGCTTCTCTGG  As – CGGCATACACAGAATACC | 2579684-2580198 | Strains of the main subspecies, 515  Other strains, 560 | –“– |
| Caucasic (–91) | 91 bp deletion in the strains of subspecies *caucasica*, phylogenetic branch 0.PE2 | S – CAAAGGGGTGCAAAGTGAC  As – GCAAGTTGTTTCAGGCCG | 467244-467428 | Strains of the subspecies *caucasica* ,94  Other strains, 185 | Current study |
| Alt(–90) | 90 bp deletion in the strains of subspecies *altaica*, phylogenetic branch 0.PE4a | S – AATGCCAGTGAGATAACCC  As – ACCTTCCTGGCCGATGTA | 1386400-1386623 | Strains of subspecies *altaica,* 134  Other strains, 224 | –“– |
| His(-205) | 205 bp deletion in the strains of subspecies *hissarica*, phylogenetic branch 0.PE4h | S – CTGACGATCGGTTTCACTTC  As – GCTTCAATTTGCTGTTTGGT | 2549643-2549773 | Strains of subspecies *hissarica,* absence –  Other strains, presence + (131) | –“– |
| Uleg(–88) | 88 bp deletion in the strains of subspecies *ulegeica*, phylogenetic branch 0.PE5 | S – ATCAATTGTAGTGGAGGGG  As – GCATCCATAGGTGCAGTA | 1646663-1646897 | Strains of subspecies *ulegeica,* 147  Other strains, 235 | –“– |
| 1 | 2 | 3 | 3 | 3 | 6 |
| Tal(-72) | 72 bp deletion in the strains of *talas* subspecies, phylogenetic branch 0.PE4t | S – CGCAAGAGTTAGGGCTGGA  As – CCTAACAAGATCCCACGGC | 2714350-2714515 | Strains of *talas* subspecies, 94  Other strains, 166 | Current study |
| ara*C* | 112 bp deletion in microtus strains, phylogenetic branch 0.PE4m | S – ATGAAACCGAGGAGCCAA  As – TCAGCCAGAATCGGGGTT | 2537778-2537897 | Microtus strains*,* absence –  Other strains, presence + (120) | –“– |
| 1.ANT/  1.ORI | Presence of cusφ phage sequence in the strains of phylogenetic branch 1.ANT/1.ORI | S – GTTCTGCTCTCTGTTTGTC  As – GTAGAGATGTGTTGCCCG | 2555559-2555974 | Strains of the phylogenetic branches 1.ANT, 1.ORI +, 416  Other strains, absence – | Nikiforov et al., 2015 |
| 2.ANT/  2.MED | 70 bp deletion in the strains of phylogenetic branch 2.ANT/2.MED | S – AAGACCTTCGCCACCAGA  As – CCAGGATTCGCCGATTCA | 2801285-2801751 | Strains of the phylogenetic branches 2.ANT. 2.MED, 397  Other strains, 467 | –“– |
| 4.ANT | Presence of pTP33 plasmid in the strains of phylogenetic branch 4.ANT | S – CGCCGCCCATTCCGATTTA  As – TCTGGCTTCCCGTTCCTGC | 3153-3312 in pTP33 plasmid (access number No KTO20860.1, NCBI GenBank) | Strains of the phylogenetic branch 4.ANT +, 160  Other strains, absence – | Current study |

* Access Number NC_003143.1 NCBI GenBank

**Table S3*B*. DNA targets and primers used for SNP typing of *Y. pestis* strains**

| **DNA target, position of marker SNP in the genome of *Y.  pestis*С092*** | **Sequences of primers**  **5’→3’** |  | **Phylogenetic branches** | | | | | | | | | | | | |
| --- | --- | --- | --- | --- | --- | --- | --- | --- | --- | --- | --- | --- | --- | --- | --- |
|  |  | **0.ANT1** | **0.ANT1** | **0.ANT2** | **0.ANT2** | **0.ANT3** | **0.ANT5** | **0.ANT5** | **2.ANT3** | **3.ANT1** | **3.ANT2** | **4.ANT** | **2.MED1** | **2.MED2** | **2.MED3** |
| YPO1404,  1587996 | S – TTCAGGAGCAACTGGAAGAA  As –TCCGGGCTGAGGTAGAGAT | T | C | C | C | C | C | C | C | C | C | C | C | C | C |
| YPO2011,  2283467 | S – CAGGCGGTTCATTGCTTT  As – CCGCTTACGCATCGGTATT | T | C | T | T | T | T | T | T | T | T | T | T | T | T |
| YPO0400,  418282 | S – ATTATGCGGTCGATCTTTTG  As – TGATACCAGAGCCGTCAGTT | G | G | T | G | G | G | G | G | G | G | G | G | G | G |
| YPO1545,  1760302 | S –CCATGCACGAAGTAGAAATAGG  As – AACGCGAGCTTATCGAACTG | T | T | T | G | T | T | T | T | T | T | T | T | T | T |
| YPO1758,  2003542 | S –TCAGCCAGCATCGGAATGT  As –CGTCCCAGGTGAAAATGCT | C | C | C | C | T | C | C | C | C | C | C | C | C | C |
| YP00114,  121618 | S – ATGAACCCAGGTGGTAGCTG  As – ACGGTATCCATTCCGTTGAA | C | C | C | C | C | A | C | C | C | C | C | C | C | C |
| YP01105,  1249092 | S – CTGCCTGTGGAGAAAATGAA  As – GTACGCCTGTACCCGTATCC | G | G | G | G | G | G | A | G | G | G | G | G | G | G |
| YPO3506  3917145 | S – CCCAATCAGCCCCCTTACA  As – GCCAGATCCATCCACCACA | G | G | G | G | G | G | T | G | G | G | G | G | G | G |
| YPO2499,  2808675 | S – GCCGCGAAGTCAGTACCAG  As –ATTCTGATCATGGCCGGTGT | C | C | C | C | C | C | C | C | A | C | G | C | C | C |
| YPO1708,  1949381 | S –GGTTTCCAATACGGGCGAT  As –GCTTTGGCGGGACTTCTAA | C | C | C | C | C | C | C | C | C | T | C | C | C | C |
| YPO1418  1610851 | S – ATCCTGTTGCCACTCAACG  As –GAAGCTGTCCACAATCGCA | G | G | G | G | Gt | G | G | G | G | G | A | G | G | G |
| YPO2744,  3074598 | S – CCGTATAGCATCGCGGAAC  As –ACCACCTACGCCTTCAACA | C | C | C | C | C | C | C | C | C | C | C | T | C | C |
| YPO1299,  1458747 | S –GAAACCCAATCGCCGTGAA  As –AGGCATTAACCCACAACGC | C | C | C | C | C | C | C | C | C | C | C | C | T | C |
| YPO0652,  710418 | S – ACGTGAGCTACGGCCTTTC  As –TCCCCACGTAACCACAGC | C | C | C | C | C | C | C | C | C | C |  | C | C | T |

* Access Number NC_003143.1 NCBI GenBank
